# Supplementary material for: Sleep-dependent upscaled excitability, saturated neuroplasticity, and modulated cognition in the human brain
Source: eLife. 2022 Jun 6;11:e69308. doi: 10.7554/eLife.69308 (PMC9225005; doi:10.7554/eLife.69308)
Supplement: Supplementary file 1. [file elife-69308-supp1.docx]

**Supplementary file 1**. Baseline measurements of the cortical excitability protocols

| **Protocol** | **measurement** | **Experimental session** | |
| --- | --- | --- | --- |
|  |  | **Sufficient sleep** | **Sleep deprivation** |
| Single-pulse MEP | SI 1_mV_ (%) | 50.40 ± 9.419 | 49.633 ± 8.973 |
|  |  |  |  |
| RMT | %MSO | 40.733 ± 7.319 | 40.466 ± 7.560 |
|  |  |  |  |
| AMT | %MSO | 34.60 ± 7.308 | 34.566 ± 6.941 |
|  |  |  |  |
| I-O curve | RMT intensity MEP | 0.176 ± 0.097 | 0.260 ± 0.171 |
|  |  |  |  |
| SICI-ICF | Single-pulse MEP | 1.033 ± 0.160 | 1.073 ± 0.190 |
|  |  |  |  |
| I-wave facilitation | Single-pulse MEP | 1.052 ± 0.216 | 1.096 ± 0.154 |
|  |  |  |  |
| SAI | Single-pulse MEP | 1.081 ± 0.143 | 1.089 ± 0.137 |
|  |  |  |  |
