# Supplementary material for: Sleep-dependent upscaled excitability, saturated neuroplasticity, and modulated cognition in the human brain
Source: eLife. 2022 Jun 6;11:e69308. doi: 10.7554/eLife.69308 (PMC9225005; doi:10.7554/eLife.69308)
Supplement: Supplementary file 2. [file elife-69308-supp2.docx]

**Supplementary file 2**. Results of repeated-measures ANOVAs for control MEPs (RMT intensity MEP for I-O curve, single-pulse MEP for other protocols), SI1mV, and %MSO for RMT/AMT.

| **Protocol** | **Measurement** | **Factor** | ***df*** | ***F*** | ***p*** |
| --- | --- | --- | --- | --- | --- |
| Single-pulse MEP | SI_1mV_ | Sleep condition | 1 | 2.72 | 0.109 |
|  |  |  |  |  |  |
| RMT | %MSO | Sleep condition | 1 | 0.231 | 0.634 |
|  |  |  |  |  |  |
| AMT | %MSO | Sleep condition | 1 | 0.012 | 0.915 |
|  |  |  |  |  |  |
| I-O curve | RMT intensity MEP | Sleep condition | 1 | 5.89 | **0.022** |
|  |  |  |  |  |  |
| SICI-ICF | Single-pulse MEP | Sleep condition | 1 | 1.01 | 0.322 |
|  |  |  |  |  |  |
| I-wave facilitation | Single-pulse MEP | Sleep condition | 1 | 1.01 | 0.323 |
|  |  |  |  |  |  |
| SAI | Single-pulse MEP | Sleep condition | 1 | 0.09 | 0.766 |
|  | | | | | |
