# Supplementary material for: Sleep-dependent upscaled excitability, saturated neuroplasticity, and modulated cognition in the human brain
Source: eLife. 2022 Jun 6;11:e69308. doi: 10.7554/eLife.69308 (PMC9225005; doi:10.7554/eLife.69308)
Supplement: Supplementary file 3. [file elife-69308-supp3.docx]

**Supplementary file 3**. Baseline measurements of the neuroplasticity sessions

| **tDCS** | **Sleep condition** | **baseline MEP** | | **SI_1mV_ (%)** | |
| --- | --- | --- | --- | --- | --- |
|  |  | **Active** | **sham** | **Active** | **sham** |
| Anodal stimulation | sufficient sleep | 1.002±0.071 | 1.019±0.071 | 48.666±8.050 | 48.533±7.827 |
|  | sleep deprivation | 1.020±0.081 | 1.026±0.068 | 46.80 ±6.667 | 46.90±6.644 |
|  |  |  |  |  |  |
| Cathodal stimulation | sufficient sleep | 1.027±0.037 | 1.005±0.065 | 51.333±10.244 | 50.866±10.183 |
|  | sleep deprivation | 1.001±0.088 | 1.018±0.056 | 50.566±10.051 | 50.133±10.043 |
|  |  |  |  |  |  |
|  | | | | | |
