# Supplementary material for: Sleep-dependent upscaled excitability, saturated neuroplasticity, and modulated cognition in the human brain
Source: eLife. 2022 Jun 6;11:e69308. doi: 10.7554/eLife.69308 (PMC9225005; doi:10.7554/eLife.69308)
Supplement: Supplementary file 4. — Data are presented as mean ± SD. [file elife-69308-supp4.docx]

**Supplementary file 4**. Reported side effects of tDCS during stimulation after sufficient sleep and sleep deprivation

| **Side effects** | **Sleep condition** | | **Anodal-tDCS** | **Sham-tDCS** | **Cathodal-tDCS** | **Sham-tDCS** |
| --- | --- | --- | --- | --- | --- | --- |
| Visual | | sufficient sleep | 0.00±0.00 | 0.00±0.00 | 0.00±0.00 | 0.00±0.00 |
|  | | sleep deprivation | 0.20±0.77 | 0.20±0.56 | 0.06±0.25 | 0.06±0.25 |
|  | |  |  |  |  |  |
| Itching | | sufficient sleep | 1.66±1.67 | 0.86±1.18 | 1.40±1.18 | 0.93±0.96 |
|  | | sleep deprivation | 2.26±1.57 | 1.26±1.43 | 1.66±1.58 | 0.93±1.16 |
|  | |  |  |  |  |  |
| Tingling | | sufficient sleep | 1.20±1.37 | 1.06±1.09 | 1.40±0.98 | 0.93±1.09 |
|  | | sleep deprivation | 1.46±1.12 | 1.00±0.92 | 1.20±1.26 | 1.00±1.25 |
|  | |  |  |  |  |  |
| Burning | | sufficient sleep | 1.26±1.43 | 1.26±1.16 | 0.73±0.96 | 0.73±1.27 |
|  | | sleep deprivation | 1.53±1.64 | 1.40±1.50 | 1.46±1.69 | 1.00±1.25 |
|  | |  |  |  |  |  |
| Pain | | sufficient sleep | 0.20±0.77 | 0.20±0.56 | 0.13±0.35 | 0.66±1.39 |
|  | | sleep deprivation | 0.46±1.24 | 0.26±0.70 | 0.60±1.05 | 0.33±0.61 |
