# Supplementary material for: Sleep-dependent upscaled excitability, saturated neuroplasticity, and modulated cognition in the human brain
Source: eLife. 2022 Jun 6;11:e69308. doi: 10.7554/eLife.69308 (PMC9225005; doi:10.7554/eLife.69308)
Supplement: Supplementary file 5. — Pairwise comparisons are calculated using Student’s t-test. n=30 (15 per group). [file elife-69308-supp5.docx]

**Supplementary file 5**. Repeated-measures ANOVA results for the presence and intensity of reported tDCS side-effects

| **Side effects** | ***Source*** | ***df*** | ***F*** | ***p*** | ***η*p^2^** |
| --- | --- | --- | --- | --- | --- |
| Visual phenomenon | sleep condition | 1 | 3.89 | 0.058 | 0.122 |
|  | group | 1 | 0.97 | 0.332 | 0.034 |
|  | tDCS state | 1 | 0.00 | 1.000 | 0.000 |
|  | sleep condition × group | 1 | 0.97 | 0.33 | 0.034 |
|  | group × tDCS state | 1 | 0.00 | 1.000 | 0.000 |
|  | sleep condition × tDCS state | 1 | 0.00 | 1.000 | 0.000 |
|  |  |  |  |  |  |
| Itching | sleep condition | 1 | 2.23 | 0.146 | 0.074 |
|  | group | 1 | 0.57 | 0.453 | 0.020 |
|  | tDCS state | 1 | 15.14 | **0.001** | 0.351 |
|  | sleep condition × group | 1 | 0.75 | 0.394 | 0.026 |
|  | group × tDCS state | 1 | 0.60 | 0.443 | 0.021 |
|  | sleep condition × tDCS state | 1 | 0.47 | 0.499 | 0.017 |
|  |  |  |  |  |  |
| Tingling | sleep condition | 1 | 0.01 | 0.924 | 0. 001 |
|  | group | 1 | 0.02 | 0.870 | 0.001 |
|  | tDCS state | 1 | 4.38 | **0.046** | 0.135 |
|  | sleep condition × group | 1 | 0.23 | 0.633 | 0.008 |
|  | group × tDCS state | 1 | 0.01 | 0.913 | 0.001 |
|  | sleep condition × tDCS state | 1 | 0.01 | 0.926 | 0.001 |
|  |  |  |  |  |  |
| Burning | sleep condition | 1 | 2.95 | 0.141 | 0.076 |
|  | group | 1 | 1.06 | 0.311 | 0.037 |
|  | tDCS state | 1 | 1.60 | 0.190 | 0.050 |
|  | sleep condition × group | 1 | 0.36 | 0.549 | 0.013 |
|  | group × tDCS state | 1 | 0.18 | 0.668 | 0.007 |
|  | sleep condition × tDCS state | 1 | 0.44 | 0.512 | 0.16 |
|  |  |  |  |  |  |
| Pain | sleep condition | 1 | 0.59 | 0.448 | 0.021 |
|  | group | 1 | 0.41 | 0.525 | 0.015 |
|  | tDCS state | 1 | 0.02 | 0.892 | 0.001 |
|  | sleep condition × group | 1 | 0.10 | 0.744 | 0.004 |
|  | group × tDCS state | 1 | 0.92 | 0.344 | 0.032 |
|  | sleep condition × tDCS state | 1 | 3.69 | 0.065 | 0.117 |
